# Novel dynamic corneal response parameters in a practice use: a critical review

**DOI:** 10.1186/s12938-019-0636-3

**Published:** 2019-02-13

**Authors:** Magdalena Jędzierowska, Robert Koprowski

**Affiliations:** Department of Biomedical Computer Systems, Faculty of Computer Science and Materials Science, Institute of Computer Science, University of Silesia, ul. Będzińska 39, 41-200 Sosnowiec, Poland

**Keywords:** Corvis tonometer, Corneal deformation, Dynamic corneal response parameters, Biomechanics

## Abstract

**Background:**

Non-contact tonometers based on the method using air puff and Scheimpflug’s fast camera are one of the latest devices allowing the measurement of intraocular pressure and additional biomechanical parameters of the cornea. Biomechanical features significantly affect changes in intraocular pressure values, as well as their changes, may indicate the possibility of corneal ectasia. This work presents the latest and already known biomechanical parameters available in the new offered software. The authors focused on their practical application and the diagnostic credibility indicated in the literature.

**Discussion:**

An overview of available literature indicates the importance of new dynamic corneal parameters. The latest parameters developed on the basis of biomechanics analysis of corneal deformation process, available in non-contact tonometers using Scheimpflug’s fast camera, are used in the evaluation of laser refractive surgery procedures, e.g. LASIK procedure. In addition, the assessment of changes in biomechanically corrected intraocular pressure confirms its independence from changes in the corneal biomechanics which may allow an intraocular pressure real assessment. The newly developed Corvis Biomechanical Index combined with the corneal tomography and topography assessment is an important aid in the classification of patients with keratoconus.

**Conclusion:**

New parameters characterising corneal deformation, including Corvis Biomechanical Index and biomechanical compensated intraocular pressure, significantly extend the diagnostic capabilities of this device and may be helpful in assessing corneal diseases of the eye. Nevertheless, further research is needed to confirm their diagnostic pertinence.

## Introduction

The significance of changing the biomechanical parameters of the cornea as well as the entire eyeball in the disease states has been repeatedly indicated in the literature [1–9]. Pathologies such as: keratoconus, glaucoma, Fuchs dystrophy and others can lead to serious disorders in this area [10–21]. The most significant is also the sensitivity of biomechanical parameters of the eye to the progression of corneal ectasia, which may be an important indicator when planning refractive procedures and assessing the possibility of corneal ectasia based on the observed changes in corneal biomechanics [22–24]. Biomechanical features and their changes are revealed during dynamic corneal changes that occur, e.g. during tonometer examination. Without any doubts, currently, the leading equipment allowing the assessment of corneal biomechanics are tonometers: ORA (Ocular Response Analyzer, Reichert Ophthalmic Instruments, Depew, NY) and introduced 5 years later (in 2010) tonometer Corvis ST (OCULUS Optikgeräte GmbH, Wetzlar, Germany). According to PubMed publication database from 2014 262 publications were published (most in 2016) in the subject of the ORA tonometer (search for the password: Ocular Response Analyzer). The search for the slogan: Corvis ended with the result of 159 publications, mostly published in 2017.

Non-contact tonometer ORA was the first to emphasise the importance of biomechanics in measuring intraocular pressure. The main difference compared to traditional air-puff tonometers is the registration of the corneal deformation process by an electro-optical infrared radiation (IR) sensor [16, 25]. The signal received by the detector is shown on the graph, along with the course of variable pressure causing corneal deformation. The characteristic moments observed in the graph of the signal recorded by the IR radiation detector are two peaks, which correspond to the moments of the first and second corneal applanation. The two basic biomechanical corneal parameters available in this device are corneal hysteresis (CH) and corneal resistance factor (CRF) [26–31]. It is worth noting that the measurement of corneal deformation in the ORA tonometer is based only on the recording of the infrared light signal.

In the Corvis ST tonometer, the basics of biomechanical parameters measurements are based on the analysis of the dynamic corneal deformation recorded by the ultra-fast Scheimpflug camera. Thanks to the possibility of reconstructing the full process of corneal deformation and observation of its dynamic changes in the video, the Corvis ST tonometer allows to obtain a much bigger number of quantitative parameters that are the basis for the description of biomechanical changes during the examination [32–36]. Various methods of image analysis and processing [37–39], or artificial intelligence methods and others [40–42] can be applied for this purpose.

The primary purpose of both devices is undoubtedly the measurement of intraocular pressure (IOP), the changes of which are the first indicator evidentiary of visual impairment, such as glaucoma or others. However, it is now known that IOP values strongly depend on the central corneal thickness (CCT), age, and in the broad sense, biomechanical parameters of the cornea and the entire eyeball [43–49]. Both ORA and Corvis ST in their software contain algorithms allowing to determine the so-called corrected IOP value. The principle of the mentioned correction is different in each of the devices. In the ORA tonometer, it is a corneal compensated IOP (IOPcc), in which the IOP value from CCT as the main factor responsible for stiffness of the cornea and based on the pressure values at the moments of the first and second applanation [16]. For the Corvis ST tonometer, a biomechanically corrected IOP (bIOP) was developed. Its value is determined on the grounds of an algorithm based on numerical simulations of dynamic corneal deformation, also taking into account the effect of variable corneal thickness [50, 51].

In this work, we will present the latest and already known biomechanical parameters possible to be obtained from tonometers using air puff. The authors will focus on their practical application and the diagnostic credibility indicated in the literature. The main aim of the work is, therefore, to review the latest literature about new dynamic corneal response (DCR) parameters and to mark their role in the clinical practice.

It should be noted that the presented article concerns all devices used in optometric medicine, using a puff of air and an ultra fast camera, allowing to record several hundred frames per second, intended for intraocular pressure measurement. Due to the widespread use of the Corvis ST tonometer both in ophthalmic surgeries and in numerous scientific articles, it will be the basis for further consideration. It should be emphasized here, that this is only an example device which allows to acquire the discussed attributes.

## The operation fundamentals of tonometers using air puff

The combination of classic measurement of intraocular pressure with the imaging of the anterior segment of the eye turned out to be a kind of breakthrough in the discussion about eyeball biomechanics. These two measurements integration was used in the tonometer using air puff and fast Scheimpflug camera. This device records the full corneal deformation process when measuring intraocular pressure using the contactless method. Thanks to the use of the ultra-fast Scheimpflug camera, it is possible to observe the behaviour of the front eye segments visible on the recorded cross-sections. The whole process is recorded in the form of a video film, it is also possible to export a series of 140 images showing corneal deformation. The Scheimpflug camera covers the cornea profile with a width of 8.5 mm, and the study itself takes only 33 ms. The force of the air pulse causing deformation is normalised and equal during each measuring, its maximum value can reach 25 kPa [52, 53].

Since 2010, in which a non-contact tonometer Covis ST was introduced, the dependence of available parameters on specific disease entities, as well as changes in their value after performed surgical procedures, was investigated. The versions of tonometer software have been changed several times, supplemented with new parameters and functionalities of the device, in the field of assessment of the biomechanical parameters impact on the obtained results, mainly the intraocular pressure value [54–60]. Biomechanical parameters of the cornea can also be measured by other types of devices used in ophthalmology using a puff of air and a fast camera that enables recording several hundred frames per second. This type of device must be enriched with software to detect the contour of the external and internal deformed cornea.

Currently, one of the most important challenges is to develop standardised values for the parameters obtained, so as to enable, among other things, early detection of ectatic corneal diseases. So far, a special indicator, called corneal biomechanical index (CBI) was developed. It indicates in an intuitive way the probability of corneal ectasia of the examined patient [61]. All of the available parameters, in the literature determined as dynamic corneal response (DCR) parameters, were isolated as a result of the specific stages of dynamic corneal deformation analysis.

### The corneal deformation-dynamic corneal response (DCR) parameters

There are nine described stages of the dynamic corneal deformation: the initial stage, preceding the deformation, then the ingoing stage in which the cornea deforms inward while maintaining its convex shape, the next stage is the moment of the first applanation or the so-called flattening of the cornea after which there is the ingoing phase of concave, where the cornea deforms and takes on a concave shape. The next moment is the so-called stage of oscillation including the moment of maximum corneal bending. At this point, we can observe the greatest vibrations, i.e. corneal oscillations. The next stage is the outgoing concave phase, when the cornea begins to return to its original shape but still retains the concave form, then the stage of the second corneal applanation, followed by the outgoing convex phase, until the cornea returns to its original shape, in which we observe the last stage, called the stage after corneal deflection [62].

Analysing the above phases in detail, a number of parameters determined by devices using air puff and Scheimpflug’s fast camera are described below.

**Fig. 1:**
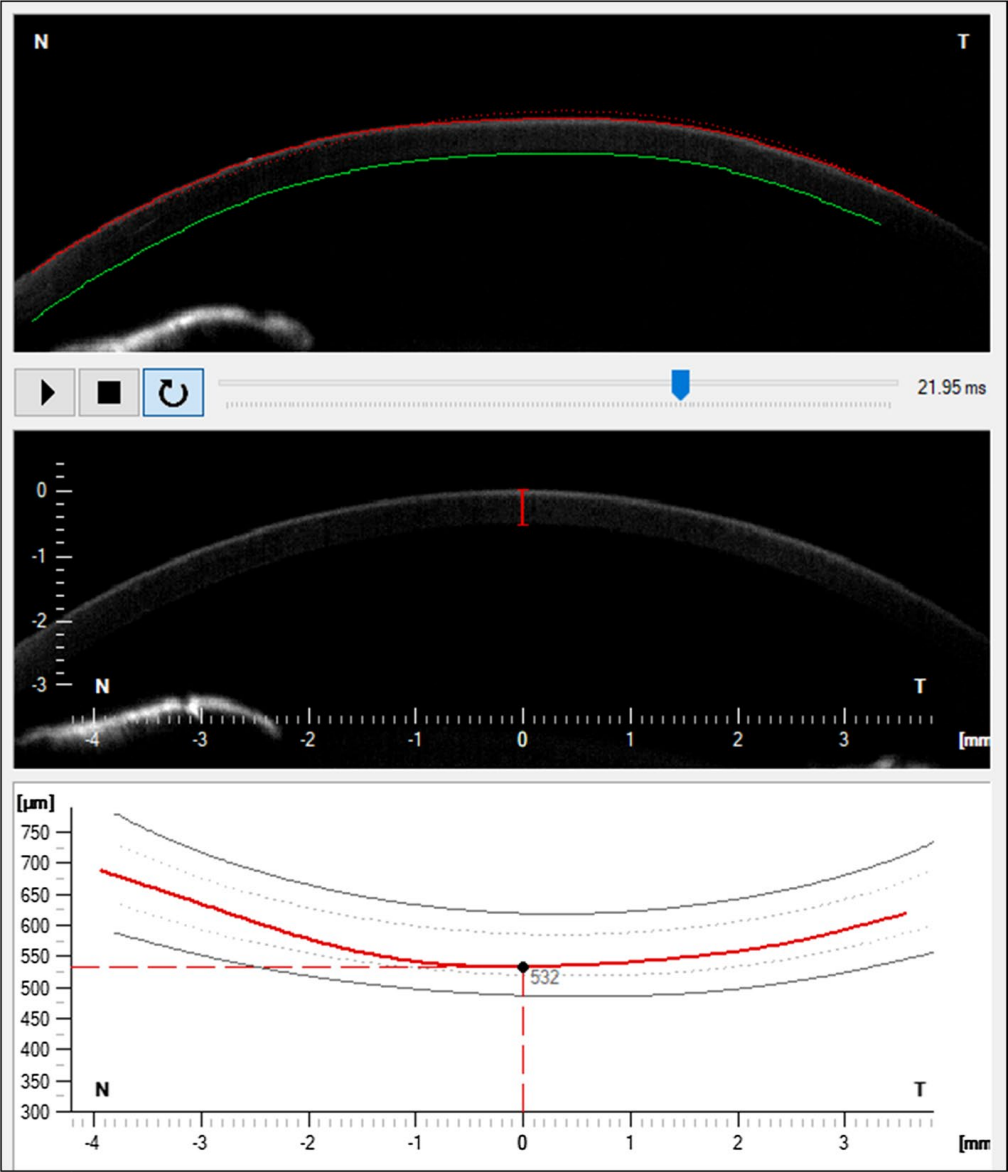
Part of the IOP/Pachy display presenting the possibility of corneal thickness measurement

The Scheimpflug camera starts acquisition of the deformation process when the air pulse is triggered. During this starting point, the cornea is in its natural, convex shape, then the pachymetry is measured—not only in the central part of the cornea (central corneal thickness CCT) but over the entire length of the cornea (Fig. 1). This allows an assessment of changes in the structure of the cornea, fluctuations in its thickness and possible asymmetry that may indicate changes in the cornea.

Then, as the air pulse pressure increases, the cornea deforms inward through the first applanation stage. At this point, the device measures the length of the first applanation segment (A1 length), the time of applanation (A1 time), the velocity of the corneal apex (A1 velocity) as well as the measurement of the corneal deflection amplitude (A1 DeflAmp). The next two parameters are: deformation amplitude ratio at 2 mm [DA ratio (2 mm)] and deflection amplitude ratio at 2 mm [DefA ratio (2 mm)]. Then cornea changes its shape from convex to concave, approaching the oscillatory phase—during this time the device measures stiffness coefficients. Respectively stiffness parameters A1 (SP-A1) and HC (SP-HC). At the moment of the of the highest corneal concavity (HC) (stage of oscillation) the parameters are determined: time of occurrence of the highest corneal concavity moment (HC time), radius of corneal curvature at its highest concavity (HC radius), maximum deformation amplitude (DA), distance between two corneal peaks during its maximum bending (peak distance) and the maximum value of the inverse of the radius of corneal curvature at the moment of the highest concavity (InvRadMax). In addition, we can obtain further indicators: corneal deflection amplitude (HCDeflAmp), corneal deflection area (HCDeflArea) as well as the difference in arc length of the outer corneal edge between the initial state of the cornea and its length at the moment of the highest concavity, measured on each side, in the horizontal plane at a distance 3.5 mm from the corneal apex (HCdArcLength) [51]. One of the last stages is the moment of the second applanation, in which the length of the second applanation (A2 length), the time of the second applanation (A2 time) and the velocity of the cornea (A2 velocity) are measured, respectively. After the examination, the corneal position can be compared during the rest period before and after deformation. As observed after such “imposition” of images, a visible displacement of the cornea in the vertical plane is observed. The value of this displacement is described by the following parameter: maximum whole eye movement (WEMax) [62, 63]. Table 1 presents, using the Corvis ST tonometer as an example, a summary of the parameters that can be obtained by analysing the corneal deformation process in non-contact tonometers equipped with a fast camera.

**Table 1:** Parameters that can be obtained from a device using an air puff and the Scheimpflug’s fast camera (using the example of the Corvis ST tonometer)

Parameter	Deformation phase	Description
A1 length (mm)	A1	Length of the flattened cornea at the first applanation
A1 velocity (mm/ms)		Velocity of the corneal apex during the first applanation
A1 time (ms)		Time from the measurement beginning to the first applanation moment
A1 DeflAmp (mm)		Corneal deflection amplitude during the first applanation, determined as the displacement of the corneal apex in relation to the initial state without the whole eye movement
DA ratio (2 mm)	Near A1	Deformation amplitude ratio at 2 mm
DefA ratio (2 mm)		Deflection amplitude ratio at 2 mm
SP-A1		Stiffness parameter A1
SP-HC		Stiffness parameter HC
DA (mm)	HC	Maximum deformation amplitude (measured at the moment of the highest corneal concavity). It is the actual sum of corneal deflection amplitude and whole eye movement
HC time (ms)		Time from the measurement beginning to the moment of reaching the highest concavity
HCDeflAmp (mm)		Corneal deflection amplitude at the moment of the highest corneal concavity
HCDeflArea (mm^2^)		Highest concavity deflection area
HCdArclength (mm)		Highest concavity delta arc length
Peak distance (mm)		Distance between the corneal peaks at the moment of the highest corneal concavity
HC radius (mm)		Radius of corneal curvature during the moment of its highest concavity
InvRadMax (1/mm)		Maximum inverse radius
WEMmax (mm)	A2	Maximum whole eye movement
A2 length (mm)		Length of the flattened cornea at the second applanation
A2 velocity (mm/ms)		Velocity of the corneal apex during the second applanation
A2 time (ms)		Time from the measurement beginning to the second applanation moment

A notable change in the last parameter adjustment is the separation of two types of parameters that are determined by taking into account (or not) the movement of the eyeball during the examination (whole eye movement). The first group is defined as deformation parameters that include WEM. However, parameters containing only “raw” corneal displacement are referred to as deflection parameters—not affected by eyeball displacement.

The repeatability of the first key parameters has been studied many times in [53, 64–66]. As one of the first Nemeth et al. [64] presented results confirming high repeatability for IOP and CCT measurements (the study group included 75 healthy eyes). The intraclass correlation coefficient (ICC) was 0.865 for IOP and 0.970 for CCT, respectively. For the amplitude of deformation (DA) and the time of the first applanation (A1 time), ICCs were obtained at 0.758 and 0.784, respectively. High repeatability of IOP measurements was also observed by Hong et al. [53] (ICC at 0.90), the ICC for reproducibility was 0.78 (for a group of 23 healthy patients and 36 patients with glaucoma). One of the basic, most often analysed parameter is the amplitude of deformation (DA). The decrease in the amplitude of deformation was associated with the increase in IOP and the greater central corneal thickness (CCT) [67–69]. An increase in the amplitude of deformation (DA) for patients with keratoconus as compared to the control group was also shown [34, 70]. In works [65, 71], there was also an increase in DA for glaucoma patients. Unfortunately, these changes were often small and the authors pointed to the need for further research and analysis of the usefulness of the parameters used. So far, no “gold standard” has been developed in this area, indicating that the value of a given parameter has significantly increased the probability of occurrence of a given eye pathology.

## Corvis Biomechanical Index (CBI)

An attempt to standardise the available biomechanical parameters is the development of the mentioned Corvis Biomechanical Index (CBI). In his work Vinciguerra et al. [61] indicate that the CBI value at 0.5 and higher indicates an increase in the possibility of corneal ectasia for the patient under study. Accuracy of this coefficient was checked in [72], for a group of 312 patients with healthy cornea and 118 patients with keratoconus (asymmetric ectasia). The area under the ROC curve for the CBI factor for the detection of corneal ectasia and the susceptibility to its occurrence was 0.864, and the sensitivity and specificity values were 70.7% and 93.3%, respectively. Significantly better results (AUC 0.988, sensitivity 94.4% and specificity 94.9%) were obtained for the Tomographic and Biomechanical Index (TBI) available through corneal tomography. Similar observations were described in earlier work [73], where 480 patients with normal cornea and 204 with keratoconus were collected. In this work, the randomised method with leave-one-out cross-validation was chosen as the best of the tested verification methods. The area under receiver operating characteristic curve (AUARC) values for the detection of corneal ectasia were 0.996 for TBI and 0.936 for CBI. Application of Corvis Biomechanical Index for the subclinical form of the keratoconus, where the patients had a confirmed diagnosis of ectasia on one eye, while the second eye was characterised by normal results of the tomography and topometry was analysed in the article [74]. In the group of 12 patients with this disease, the CBI index values for the eye with correct (indicative of no disease) results obtained from tomographic measurements and corneal topography resulted in CBI values greater than 0.5 for both eyes.

Hirasawa et al. [75] investigated the change in CBI after cataract surgery. The research group included 39 eyes with a cataract. Control examinations were carried out before surgery and week, month and 3 months after surgery. Among the parameters studied, the CBI index increased significantly after 1 week after surgery (P < 0.01), however, in the reference periods, after the 1st and 3rd month of surgery, it was at the same level as before the surgery. What is more, the mean value of the CBI before the surgery was 0.15 (less than the value indicated in [61], where the “cut-off” point for patients with keratoconus was 0.5) 1 week after surgery 0.24 and 0.15 after 3 months. Hirasawa and his team, therefore, indicate the need for a more detailed study of the usefulness of the CBI index to assess patients after cataract surgery. Obtained high values do not have to be an indicator of the keratoconus. The summary of Corvis Biomechanical Index (CBI) evaluation is presented in Table 2.

**Table 2:** Corvis Biomechanical Index (CBI) evaluation

Author	Participants	Parameter	Results
Vinciguerra et al. [61]	478 healthy patients and 180 patients with keratoconus	Corvis Biomechanical Index (CBI)	For the broad international research group, the CBI index showed statistically significant sensitivity and specificity in the classification of patients with keratoconus
Ferreira-Mendes et al. [72]	312 healthy patients and 118 patients with keratoconus	Tomographic and Biomechanical Index (TBI), CBI and Belin/Ambrósio Deviation Index (BAD-DI)	The TBI parameter showed the highest accuracy in distinguishing healthy eyes from the eyes with corneal ectasia—the AUC value for the TBI index was statistically higher than the values for CBI and BAD-DI
Ambrosio et al. [73]	480 healthy and 204 patients with keratoconus	TBI, CBI and Belin/Ambrósio Deviation (BAD-D)	The TBI index showed statistically better accuracy in the detection of corneal ectasia than CBI and BAD-D. The TBI parameter proved effective in distinguishing subclinical forms of corneal ectasia
Vinciguerra et al. [74]	12 patients with a diagnosed subclinical form of the keratoconus	CBI, tomography and topography of the cornea	In all analysed cases, an increase in the CBI index indicating changes in corneal biomechanics was demonstrated for the correct results of corneal tomography and topography
Hirasawa et al. [75]	39 patients suffering from cataract	Ten different biomechanical parameters, including CBI	There was no expected decline in the CBI index. The best results were obtained for SP-A1 (significant decrease) and for DA and integrated radius, which increased, suggesting less corneal stiffness after the procedure

In reference to the cited literature, the recently introduced Corvis Biomechanical Index (CBI) is a very prospective tool in the diagnosis of corneal ectasia. In spite of very good initial results [61] indicating high sensitivity and specificity of this parameter in the classification of patients with keratoconus, it should nowadays be an additional diagnostic tool which complements tomographic and topographic examination of the cornea and not an independent parameter which is the basis for the corneal pathology assessment. This statement is indicated by studies describing the ambiguity of the results obtained. It should be undoubtedly stated that this parameter confirms the importance of biomechanics in the diagnosis of ectatic corneal diseases, especially in cases where biomechanical analysis shows abnormalities not observed in tomographic examinations, which often occurs in subclinical cases of keratoconus.

## Dynamic corneal response (DCR) parameters

### Repeatability of the dynamic corneal response parameters

**Fig. 2:**
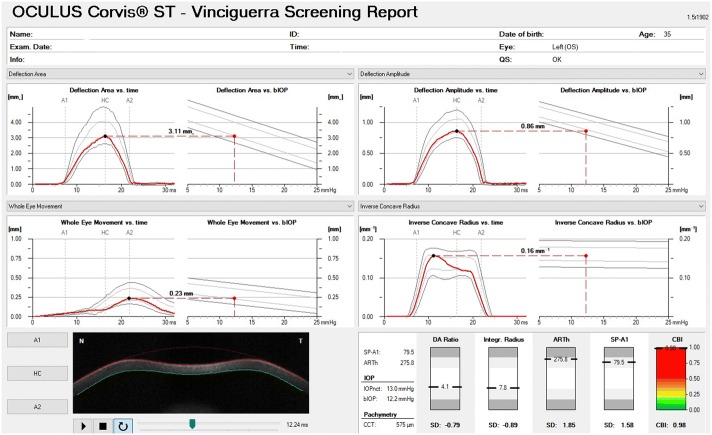
Screenshot of Corvis ST display presenting the Vinciguerra Screening Report for patient with keratoconus

Biomechanics of the cornea significantly affect the values of intraocular pressure [10, 16, 22, 25, 76–78]. This was also confirmed by the latest work analysing the correlation of new dynamic corneal response parameters (DCRs) with IOP values [51]. Therefore, not only age and central corneal thickness are parameters requiring consideration during the tests [47, 79]. In the new version of Corvis ST tonometer software in the tab containing so-called The Vinciguerra Screening Report (Fig. 2) there are two IOP values—IOPnct, i.e. uncorrected pressure (hereinafter referred to as IOP in the paper) and bIOP—i.e. biomechanically corrected IOP. bIOP was developed in its original version by the team of Joda et al. [50], whereas in the Corvis ST tonometer the reported pressure value is calculated according to the modified algorithm [51, 80].

Repeatability and reproducibility for IOP and bIOP values were first studied in Lopes et al. [81]. Tests carried out in a group of 32 healthy patients. There were also analysed eight selected DCR parameters: maximum deformation amplitude, maximum deflection amplitude, DA ratio at 2 mm, DA ratio at 1 mm, integrated radius, maximum inverse radius, the first applanation velocity and stiffness parameter at the first applanation. The results showed very good in-subject standard deviation values for repeatability and reproducibility for uncorrected pressure (IOP)—about 1 mmHg, 0.98 and 1.12, respectively. The coefficient of variation (CV) was 6.6% (repeatability) and 7.6% (reproducibility). For bIOP, these values remained at a similar level—about 1 mmHg, respectively 0.89 for repeatability and 1.05 for reproducibility. However, CV values for bOPI are 6.1% and 7.2% for repeatability and reproducibility. For the other analysed parameters, good results were also obtained—CV values for most of them were below 4% for repeatability and 6% for reproducibility. The high repeatability of DCR parameters has also been confirmed in studies [82]. Table 3 summarizes the results of measuring the repeatability of DCRs.

**Table 3:** Repeatability of dynamic corneal response parameters

Author	Participants	Parameters	Results
Lopes et al. [81]	32 healthy patients	IOP; bIOP; and eight selected DCR parameters	Corvis ST showed good repeatability and reproducibility for unadjusted IOP measurements and biomechanically corrected IOP
Miki et al. [82]	48 healthy patients	IOP and 35 DCR parameters available in the Corvis ST tonometer	Repeatability for 22 out of 35 analysed corneal deformation parameters was at a high level (the intraclass correlation coefficient ICC ≥ 0.75)

### Changes in the biomechanical parameters of the cornea after surgery

One of the main assumptions of the developed algorithm allowing the determination of biomechanical corrected IOP was the independence of IOP values from changes in corneal thickness. In the work [83]. Chen together with his research team analysed changes in bIOP, unadjusted IOP and IOP measured with Goldman’s applanation tonometer for patients before and after SMILE (small-incision lenticule extraction)—14 patients and LASIK (laser in situ keratomileusis)—22 patients. The results showed that changes in bIOP values before and after both treatments were statistically insignificant (0.1 ± 2.1 mmHg and P = 0.80 for LASIK and 0.8 ± 1.8 mmHg and P = 0.27 for SMILE). What is more, this pressure was also negligible correlated with CCT (statistical analysis according to Pearson showed P values above 0.05). Significant correlation and significant pressure drop after both treatments were observed both for unadjusted IOP and pressure measured by the Goldman apparatus.

The analysis of DCR parameters and changes in bIOP after LASIK and PKR (transepithelial photorefractive keratectomy) is presented in [44]. For 64 cases, no significant difference was found in the change of bIOP after LASIK surgery (preoperative bIOP: 16.12 ± 1.66 mmHg, bIOP after surgery 15.86 ± 1.32 mmHg) as well as PRK (preoperative biopsy: 16.30 ± 1.68 mmHg, bIOP after surgery 16.60 ± 1.29 mmHg). In addition, two of the analysed parameters significantly increased—deformation amplitude ratio at 2.0 mm and integrated inverse radius, two more characterised by a noticeable drop value—stiffness parameter at first applanation and Ambrosio relational thickness through the horizontal meridian (ARTh) [61] (P < 0.001), both after LASIK and PRK. The authors point to the fact that changes in the amplitude of deformation amplitude as well as integrated inverse radius were lower in the case of PRK, suggesting less invasive treatment in corneal biomechanics.

The research team in a similar composition [84] also analysed changes in the same DCR parameters (and a few additional ones) also after the PRK and PRK procedure combined with the LASIK procedure. Here, too, no significant changes in bIOP values were observed which is consistent with the pioneering work in this topic [51], which indicates biomechanical IOP values corrected as less sensitive to changes in corneal parameters such as CCT and age. There was a statistically significant decrease in stiffness parameters (SP-A1 and SP-HC) after both treatments and a significant increase in parameters: deformation amplitude ratio at 2.0 mm, deformation amplitude ratio at 1.0 mm and integrated inverse radius. In the case of these procedures, smaller changes in biomechanical parameters were detected after the treatment of the combined PRK and LASIK surgery.

Significant changes for the new biomechanical parameters available in the Corvis ST tonometer were noted in work [85]. For a group of 34 patients, the results of DCR parameters were compared before and after SMILE. All of the parameters analysed: corneal biomechanical index, deflection amplitude ratio at 1.0 mm, deflection amplitude ratio at 2.0 mm, integrated inverse curvature radius, stiffness parameter at A1 (SP-A1) changed significantly after the surgery was performed (P < 0.05) which remains in accordance with previous quoted works. One of the most important conclusions is the lack of correlation between the SP-A1 and CCT parameters (P = 0.15) after the SMILE procedure. As the authors themselves note, this suggests that in future works this parameter will be used as a valuable indicator of the corneal biomechanical features independent of corneal thickness. However, different results were obtained for the change of bIOP, which after the performed SMILE surgery significantly decreased. A significant difference in the values of bIOP after surgery was also noted in works [75, 86], where the change of corneal biomechanics was evaluated after cross-linking (CLX) and cataract surgery.

It is worth noting that after the CLX surgery Vinciguerra et al. [86] for the analysed parameters (after 6 months from the surgery) noted a significant increase in stiffness parameters: SP-A1 and SP-HC and a significant decrease in the values of three parameters: inverse concave radius (1/R), deflection amplitude (DefA) and deformation amplitude ratio (DA ratio)—P < 0.05 for Wilcoxon paired test. The above suggests the possibility of using these parameters as indicators allowing early assessment of the effects of cross-linking operations.

Assessing the above mentioned literature it can be stated that in clinical practice the most important are parameters related to the amplitude of deformation (deformation amplitude ratio) as well as stiffness parameters (SP-A1 and SP-HC). These parameters can be very helpful in evaluating the progression of corneal diseases and early evaluation of the performed surgical procedures effectiveness.

## Summary

The subject of the influence of biomechanical properties of the cornea and the entire eyeball on the results obtained in tonometry studies has been present in the literature for a long time. It seemed that the breakthrough in this area and the final answers will bring new devices—tonometer ORA and Corvis ST, which providing additional measurements allow the correction of assessed indicators, such as intraocular pressure. Nevertheless, it has been already 8 years since the introduction of non-contact tonometer, and the discussion in this topic is still alive, and the list of topics requiring further research is still growing. The above confirms also the literature collected for the purposes of this work. It is also worth noting that another area which is more and more often connected with Corneal Visualization Scheimpflug Technology is modelling of corneal deformation [87–95].

The basic biomechanical parameters available to date in the Corneal Visualization Scheimpflug Technology tonometer software were limited to the quantitative evaluation of certain distinct corneal features. Unfortunately, the lack of standardisation of available indicators significantly limited their use in clinical practice. An example of work on the analysis of the corneal dynamic response after surgery is shown in Table 4.

**Table 4:** Dynamic corneal response parameters after surgery

Author	Participants	Parameters	Results
Chen et al. [83]	14 patients referred for LASIK treatment and 22 patients referred for SMILE treatment	bIOP, IOP, Goldman IOP	The bIOP values before and after LASIK and SMILE treatments turned out to be slightly different and did not show any significant correlation with CCT Unlike other pressures—Goldman IOP and IOP, which showed significant changes after the treatments and a significant correlation with CCT
Hirasawa et al. [75]	39 patients qualified for cataract surgery	CBI, IOP (Goldman), bIOP and six DCR parameters	Changes in IOP values and biomechanical parameters after cataract surgery were recorded Significant decrease in SP-A1 and significant increase in DA max. after the reference period of 3 months The bIOP values showed a statistically significant decrease
Ueki et al. [96]	8 patients after LASIK (with keratectasia), 11 patients after LASIK (without keratectasia), 24 patients with keratoconus, 34 healthy patients	IOP and 10 DCR parameters	For parameters: HC radius and HCDeflAmp there was a significant difference (P < 0.05) between patients with keratectasia after LASIK and patients without keratectasia after LASIK

As can be seen from Table 4, bIOP values showed a statistically significant index enabling to distinguish between patients after LASIK and SMILE procedures. Similarly, parameters obtained from the Goldman device allow to distinguish patients qualified for cataract surgery [75, 83, 96].

The latest dynamic corneal response parameters analysed in this review, available in the updated version of the Corvis ST tonometer software are still being tested and their clinical application is being verified. The most important conclusions from the analysed literature are presented below.

1. Biomechanically corrected intraocular pressure value in most of the works is a parameter independent of corneal biomechanics. This is confirmed by studies of patients with healthy cornea as well as of patients after various refractive procedures. bIOP can be classified as a parameter independent of CCT and age. The significant changes in this parameter noted in several works [75, 85, 86] draw attention to the fact that special accuracy in the measurement of the bIOP should be kept, because other factors, which are not included in the correction algorithm, also affect the pressure value. The point are pressure fluctuations depending on the time of day, its dependence on blood pressure [97, 98], as well as the number of repetitions and the time interval between subsequent tests.
2. The Corvis Biomechanical Index available in The Vinciguerra Screening Report, confirmed its effectiveness in detecting patients with keratoconus. However, the effectiveness of CBI in detecting other corneal pathologies, e.g. cataracts, has not been confirmed. What is more, it was noted that the CBI index is more sensitive than the Tomography and Biomechanical Index (TBI), available after Corvis ST integration with corneal tomography by Pentacam. However, it is important, that the combination of both of these indicators allows better classification, which may prove to be a key element in early diagnosis and so far allows for the best results in this area.
3. The most frequently analysed dynamic corneal response parameters are: deformation amplitude ratio, deflection amplitude ratio, stiffness parameter A1, stiffness parameter HC, integrated inverse radius and Ambrosio relational thickness through the horizontal meridian. Not without significance is the fact that these parameters are one of the parts of The Vinciguerra Screening Report, which provides their absolute values and their standard deviations from the average. These parameters can be early indicators for the evaluation of the effects of refractive treatments of the cornea. Table 5 presents a list of changes of the above parameters depending on the performed procedure.

**Table 5:** List of changes in DCR parameters after corneal surgery

Parameter	Lee et al. [84]		Lee et al. [44]		Fernandez et al. [85]	Hirasawa et al. [75].	Vinciguerra et al. [86]
	PKR (n = 35)	PRK + LASIK (n = 34)	LASIK (n = 64)	PRK (n = 65)	SMILE (n = 43)	CATARACT (n = 39)	CLX (n = 34)
DA ratio (1 mm)	↑	↑	–	–	↑	↑	↓
DA ratio (2 mm)	↑	↑	↑	↑	↑	–	–
SP-A1	↓	↓	↓	↓	↓	↓	↑
SP-HC	↓	↓	–	–	–	–	↑
IntInvRad	↑	↑	↑	↑	↑	↑	↓
bIOP	No significant difference	No significant difference	No significant difference	No significant difference	↓	↓	↑
ARTh	–	–	↓	↓	–	↓	–

The up arrow (↑) indicates a significant increase in the given parameter; down arrow (↓) indicates a significant decrease in a given parameter; horizontal lines (–) indicate the lack of analysis of a given parameter

PRK: photorefractive keratectomy; LASIK: laser-assisted laser in situ keratomileusis; SMILE: small-incision lenticule extraction; CATARACR: cataract surgery; CLX: corneal cross-linking; DA ratio (1 mm): deformation amplitude ratio at 1 mm; DA ratio (2 mm): deformation amplitude ratio at 2 mm; SP-A1: stiffness parameter A1; SP-HC: stiffness parameter HC; IntInvRad: integrated inverse radius; bIOP: biomechanically corrected IOP; ARTh: Ambrosio relational thickness to the horizontal profile
4. Special attention should be paid here to the unverified relation between the actual intraocular pressure and the pressure measured by means of devices using air puff. The Goldmann Tonometer is still the gold standard for intraocular pressure measurement [99, 100].
5. Not without significance here is the progress in technology, which allows non-contact measurement as well as data transmission and recording on an external storage medium. Such modern devices designed for IOP measurement, and in the future allowing for the analysis of corneal biomechanical parameters are:
  Diaton: The portable and manual Diaton device does not need sterilization or anesthesia. As the IOP is measured through the eyelid over the sclera, there is no influence of corneal thickness or corneal stiffness [101].
  Icare: The advantage of Icare is that it is portable and it does not need sterilization or anesthesia. What is more, it may be used at home. The contact time is extremely short and is able to receive temporary drops or spikes in the IOP; in order to overcome this variability, the user has to take an average of 4 out of 6 readings. This modern tonometer records the IOP, time and date and calculates the quality result using a dedicated algorithm. Every measurement is stored in the device and may become available to the physician later [102, 103].
  Triggerfish: This modern device consists of a contact lens sensor and a small antenna near the eye that transmits data via wireless technology. One of its major advantages is that it may be used at home. It also provides permanent measurement. The 24-h IOP patterns, for example, can be monitored continuously without waking up the patient [104].
6. Very often the aspect of repeatability of measurements is omitted in the literature on biomechanical parameters measurement. It should be noted here, in particular, that at high individual variability, for different biomechanical parameters of the cornea, as well as its shape and arrangement, the measurements may differ significantly from each other. In particular, the patient, e.g. for different head positions in the chin rest, indirectly influences the obtained results. Error values vary and depend especially on the accuracy and reliability of the operator’s (ophthalmologist) work. These errors range from a few to several percents [105].

The literature presented in this review is one of the first after the introduction of new biomechanical parameters of the cornea. They present promising results, but further studies on the usefulness of these parameters are necessary. One of the biggest limitations is still a small number of analysed cases, in particular in the area of analysis of changes in parameters after surgery. However, thanks to the more and more frequent cooperation of international units, the acquired data allows the standardisation of data received and may soon bring satisfactory results. In conclusion, it can be said that thanks to such a dynamic development in the field of assessment and verification of ocular biomechanics, it is possible to better understand its impact on the genesis of eye diseases, and most importantly, it will also allow effective prevention and early detection of corneal disorders.

## Abbreviations

ORA: Ocular Response Analyzer
IOP: intraocular pressure
CCT: central corneal thickness
bIOP: biomechanically corrected intraocular pressure
DCRs: dynamic corneal response parameters
CBI: corneal biomechanical index
TBI: Tomographic and Biomechanical Index
BAD-DI: Belin/Ambrósio Deviation Index
CV: coefficient of variation
ICC: intraclass correlation coefficient
SMILE: small-incision lenticule extraction
LASIK: laser in situ keratomileusis
PKR: transepithelial photorefractive keratectomy
CLX: corneal cross-linking

## References

[1] Clement CI, Parker DGA, Goldberg I (2016). Intra-ocular pressure measurement in a patient with a thin, thick or abnormal cornea. Open Ophthalmol J.

[2] Antonios R, Fattah MA, Maalouf F (2016). Central corneal thickness after cross-linking using high-definition optical coherence tomography, ultrasound, and dual Scheimpflug tomography: a comparative study over one year. Am J Ophthalmol.

[3] Greenstein SA, Fry KL, Hersh PS (2012). In vivo biomechanical changes after corneal collagen cross-linking for keratoconus and corneal ectasia: 1-year analysis of a randomized, controlled, clinical trial. Cornea.

[4] Smedowski A, Weglarz B, Tarnawska D (2014). Comparison of three intraocular pressure measurement methods including biomechanical properties of the cornea. Invest Ophthalmol Vis Sci.

[5] Kempf R, Kurita Y, Iida Y (2006). Understanding eye deformation in non-contact tonometry. Conf Proc IEEE Eng Med Biol Soc.

[6] Piñero DP, Alcón N (2015). Corneal biomechanics: a review. Clin Exp Optom.

[7] Ambrósio R, Correia FF, Lopes B (2017). Corneal biomechanics in ectatic diseases: refractive surgery implications. Open Ophthalmol J.

[8] Lanza M, Cennamo M, Iaccarino S (2014). Evaluation of corneal deformation analyzed with scheimpflug based device in healthy eyes and diseased ones. BioMed Res Int.

[9] Aksoy D, Ortak H, Kurt S (2014). Central corneal thickness and its relationship to Parkinson’ s disease severity. Can J Ophthalmol.

[10] Kotecha A (2007). What biomechanical properties of the cornea are relevant for the clinician?. Surv Ophthalmol.

[11] Touboul D, Roberts C, Kérautret J (2008). Correlations between corneal hysteresis, intraocular pressure, and corneal central pachymetry. J Cataract Refract Surg.

[12] Krysik K, Dobrowolski D, Wroblewska-Czajka E (2018). Comparison of the techniques of secondary intraocular lens implantation after penetrating keratoplasty. J Ophthalmol.

[13] Krysik K, Dobrowolski D, Lyssek-Boron A (2017). Differences in surgical management of corneal perforations, measured over six years. J Ophthalmol.

[14] Tian L, Ko MWL, Wang L-K (2014). Assessment of ocular biomechanics using dynamic ultra high-speed scheimpflug imaging in keratoconic and normal eyes. J Refract Surg.

[15] Roy AS, Shetty R, Kummelil MK (2013). Keratoconus: a biomechanical perspective on loss of corneal stiffness. Indian J Ophthalmol.

[16] Luce DA (2005). Determining in vivo biomechanical properties of the cornea with an ocular response analyzer. J Cataract Refract Surg.

[17] Medeiros FA, Weinreb RN (2006). Evaluation of the influence of corneal biomechanical properties on intraocular pressure measurements using the ocular response analyzer. J Glaucoma.

[18] Koprowski R, Ambrósio R (2015). Quantitative assessment of corneal vibrations during intraocular pressure measurement with the air-puff method in patients with keratoconus. Comput Biol Med.

[19] Fontes BM, Ambrósio R, Velarde GC (2011). Corneal biomechanical evaluation in healthy thin corneas compared with matched keratoconus cases. Arq Bras Oftalmol.

[20] Galletti JD, Ruiseñor Vázquez PR, Fuentes Bonthoux F (2015). Multivariate analysis of the ocular response analyzer’s corneal deformation response curve for early keratoconus detection. J Ophthalmol.

[21] Ariza-Gracia M, Zurita JF, Piñero DP (2015). Coupled biomechanical response of the cornea assessed by non-contact tonometry. A simulation study. PLoS ONE.

[22] Ambrosio R, Nogueira LP, Caldas DL (2011). Evaluation of corneal shape and biomechanics before LASIK. Int Ophthalmol Clin.

[23] Shetty R, Francis M, Shroff R (2017). Corneal biomechanical changes and tissue remodeling after SMILE and LASIK. Invest Ophthalmol Vis Sci.

[24] Bekesi N, Kochevar IE, Marcos S (2016). Corneal biomechanical response following collagen cross-linking with Rose Bengal-green light and riboflavin-UVA. Invest Ophthalmol Vis Sci.

[25] Franco S, Lira M (2009). Biomechanical properties of the cornea measured by the Ocular Response Analyzer and their association with intraocular pressure and the central corneal curvature. Clin Exp Optom.

[26] Nessim M, Mollan SP, Wolffsohn JS (2013). The relationship between measurement method and corneal structure on apparent intraocular pressure in glaucoma and ocular hypertension. Cont Lens Anterior Eye.

[27] Congdon NG, Broman AT, Bandeen-Roche K (2006). Central corneal thickness and corneal hysteresis associated with glaucoma damage. Am J Ophthalmol.

[28] Kamiya K, Hagishima M, Fujimura F (2008). Factors affecting corneal hysteresis in normal eyes. Graefes Arch Clin Exp Ophthalmol.

[29] Tejwani S, Shetty R, Kurien M (2014). Biomechanics of the cornea evaluated by spectral analysis of waveforms from Ocular Response Analyzer and Corvis-ST. PLoS ONE.

[30] Pepose JS, Feigenbaum SK, Qazi MA (2007). Changes in corneal biomechanics and intraocular pressure following LASIK using static, dynamic, and noncontact tonometry. Am J Ophthalmol.

[31] Lam AKC, Chen D, Tse J (2010). The usefulness of waveform score from the ocular response analyzer. Optom Vis Sci.

[32] Jedzierowska M, Koprowski R, Wrobel Z (2014). Overview of the ocular biomechanical properties measured by the Ocular Response Analyzer and the Corvis ST. Inf Technol Biomed.

[33] Koprowski R (2014). Automatic method of analysis and measurement of additional parameters of corneal deformation in the Corvis tonometer. Biomed Eng Online.

[34] Ji C, Yu J, Li T (2015). Dynamic curvature topography for evaluating the anterior corneal surface change with Corvis ST. Biomed Eng Online.

[35] Koprowski R, Ambrósio R, Reisdorf S (2015). Scheimpflug camera in the quantitative assessment of reproducibility of high-speed corneal deformation during intraocular pressure measurement. J Biophotonics.

[36] Jankowska-Szmul J, Dobrowolski D, Krysik K (2016). Changes in technique and indications for keratoplasty in Poland, 1989 to 2014: an analysis of corneal transplantations performed at Saint Barbara Hospital, Trauma Center, Sosnowiec, Poland. Transplant Proc.

[37] Popielski P, Koprowski R, Wróbel Z (2018). The matching method for veins images. Comput Med Imaging Graph.

[38] Wójcicka A, Jędrusik P, Stolarz M, Pięketka E, Kawa J, Wieclawek W (2014). Using analysis algorithms and image processing for quantitative description of colon cancer cells. Information technologies in biomedicine.

[39] Walczak M, Pięketka E, Badura P, Kawa J (2016). 3D measurement of geometrical distortion of synchrotron-based perforated polymer with Matlab algorithm. Information technologies in medicine: 5th international conference.

[40] Glowacz A, Glowacz Z (2016). Diagnostics of stator faults of the single-phase induction motor using thermal images, MoASoS and selected classifiers. Measurement.

[41] Glowacz A, Glowacz Z (2016). Recognition of images of finger skin with application of histogram, image filtration and K-NN classifier. Biocybern Biomed Eng.

[42] Glowacz A, Glowacz A, Glowacz Z (2015). Recognition of thermal images of direct current motor with application of area perimeter vector and Bayes classifier. Meas Sci Rev.

[43] Jędzierowska M, Koprowski R, Wróbel Z, Pietka E, Badura P, Kawa J (2019). Limitations of corneal deformation modelling during IOP measurement—a review. Information technology in biomedicine. ITIB 2018.

[44] Lee H, Roberts CJ, Kim T (2017). Changes in biomechanically corrected intraocular pressure and dynamic corneal response parameters before and after transepithelial photorefractive keratectomy and femtosecond laser-assisted laser in situ keratomileusis. J Cartact Refract Surg.

[45] Boszczyk A, Kasprzak H, Agnieszka J (2017). Eye retraction and rotation during Corvis ST ‘air puff’ intraocular pressure measurement and its quantitative analysis. Ophthalmic Physiol Optics.

[46] Kotecha A, Elsheikh A, Roberts CR (2006). Corneal thickness- and age-related biomechanical properties of the cornea measured with the ocular response analyzer. Invest Ophthalmol Vis Sci.

[47] Kotecha A, White ET, Shewry JM (2005). The relative effects of corneal thickness and age on Goldmann applanation tonometry and dynamic contour tonometry. Br J Ophthalmol.

[48] Wang AS, Alencar LM, Weinreb RN (2013). Repeatability and reproducibility of Goldmann applanation, dynamic contour, and ocular response analyzer tonometry. J Glaucoma.

[49] Krysik K, Dobrowolski D, Polanowska K (2017). Measurements of corneal thickness in eyes with pseudoexfoliation syndrome : comparative study of different image processing protocols. J Healthc Eng.

[50] Joda AA, Shervin MMS, Kook D (2016). Development and validation of a correction equation for Corvis tonometry. Comput Methods Biomech Biomed Eng.

[51] Vinciguerra R, Elsheikh A, Roberts CJ (2016). Influence of pachymetry and intraocular pressure on dynamic corneal response parameters in healthy patients. J Refract Surg.

[52] Ambrósio R, Ramos I, Luz A (2013). Dynamic ultra high speed Scheimpflug imaging for assessing corneal biomechanical properties. Rev Bras Oftalmol.

[53] Hong J, Xu J, Wei A (2013). A new tonometer—the Corvis ST tonometer: clinical comparison with noncontact and Goldmann applanation tonometers. Invest Ophthalmol Vis Sci.

[54] Rogowska ME, Iskander DR (2015). Age-related changes in corneal deformation dynamics utilizing Scheimpflug imaging. PLoS ONE.

[55] Nakao Y, Kiuchi Y, Okimoto S (2017). A comparison of the corrected intraocular pressure obtained by the corvis ST and reichert 7CR tonometers in glaucoma patients. PLoS One.

[56] Faria-Correia F, Ambrósio R (2016). Clinical applications of the Scheimpflug principle in ophthalmology. Rev Bras Oftalmol.

[57] Roberts C. Two novel stiffness parameters for the Corvis ST. OCULUS Special Supplement. 2016.

[58] Koprowski R (2015). Open source software for the analysis of corneal deformation parameters on the images from the Corvis tonometer. Biomed Eng Online.

[59] Eliasy A, Chen K, Vinciguerra R (2018). Ex-vivo experimental validation of biomechanically-corrected intraocular pressure measurements on human eyes using the CorVis ST. Exp Eye Res.

[60] Krysik K, Wroblewska-Czajka E, Lyssek-Boron A (2018). Total penetrating keratoplasty : indications, therapeutic approach, and long-term follow-up. J Ophthalmol.

[61] Vinciguerra R, Ambrósio R, Elsheikh A (2016). Detection of keratoconus with a new biomechanical index. J Refract Surg.

[62] Roberts CJ, Mahmoud AM, Bons JP (2017). Introduction of two novel stiffness parameters and interpretation of air puff-induced biomechanical deformation parameters with a dynamic Scheimpflug analyzer. J Refract Surg.

[63] Miki A, Maeda N, Ikuno Y (2017). Factors associated with corneal deformation responses measured with a dynamic Scheimpflug analyzer. Invest Ophthalmol Vis Sci.

[64] Nemeth G, Hassan Z, Csutak A (2013). Repeatability of ocular biomechanical data measurements with a Scheimpflug-based noncontact device on normal corneas. J Refract Surg.

[65] Ali NQ, Patel DV, McGhee CNJ (2014). Biomechanical responses of healthy and keratoconic corneas measured using a noncontact Scheimpflug-based tonometer. Invest Ophthalmol Vis Sci.

[66] Bak-Nielsen S, Pedersen I, Ivarsen A (2015). Repeatability, reproducibility, and age dependency of dynamic Scheimpflug-based pneumotonometer and its correlation with a dynamic bidirectional pneumotonometry device. Cornea.

[67] Hon Y, Lam AKC (2013). Corneal deformation measurement using Scheimpflug noncontact tonometry. Optom Vis Sci.

[68] Tian L, Huang Y, Wang L (2014). Corneal biomechanical assessment using corneal visualization Scheimpflug technology in keratoconic and normal eyes. J Ophthalmol.

[69] Kling S, Marcos S (2013). Contributing factors to corneal deformation in air puff measurements. Invest Ophthalmol Vis Sci.

[70] Ye C, Yu M, Lai G (2015). Variability of corneal deformation response in normal and keratoconic eyes. Optom Vis Sci.

[71] Jung Y, Park HL, Yang HJ (2017). Characteristics of corneal biomechanical responses detected by a non-contact Scheimpflug-based tonometer in eyes with glaucoma. Acta Ophthalmol.

[72] Ferreira-Mendes J, Lopes B, Faria-Correia F (2018). Enhanced ectasia detection using corneal tomography and biomechanics. Am J Ophthalmol.

[73] Ambrósio R, Lopes BT, Faria-correia F (2017). Integration of Scheimpflug-based corneal tomography and biomechanical assessments for enhancing ectasia detection. J Refract Surg.

[74] Vinciguerra R, Ambrósio R, Roberts CJ (2017). Biomechanical characterization of subclinical keratoconus without topographic or tomographic abnormalities. J Refract Surg.

[75] Hirasawa K, Nakakura S, Nakao Y (2018). Changes in corneal biomechanics and intraocular pressure following cataract surgery. Am J Ophthalmol.

[76] Ogbuehi KC, Osuagwu UL (2013). Corneal biomechanical properties: precision and influence on tonometry. Cont Lens Anterior Eye.

[77] Liu J, Roberts CJ (2005). Influence of corneal biomechanical properties on intraocular pressure measurement: quantitative analysis. J Cataract Refract Surg.

[78] Lyssek-Boroń A, Wylęgała A, Polanowska K (2017). Longitudinal changes in retinal nerve fiber layer thickness evaluated using avanti Rtvue-XR optical coherence tomography after 23G vitrectomy for epiretinal membrane in patients with open-angle glaucoma. J Healthc Eng.

[79] Doughty MJ, Zaman ML (2000). Human corneal thickness and its impact on intraocular pressure measures: a review and meta-analysis approach. Surv Ophthalmol.

[80] Elsheikh A (2010). Finite element modeling of corneal biomechanical behavior. J Refract Surg.

[81] Lopes BT, Roberts CJ, Elsheikh A (2017). Repeatability and reproducibility of intraocular pressure and dynamic corneal response parameters assessed by the Corvis ST. J Ophthalmol.

[82] Miki A, Maeda N, Asai T (2017). Measurement repeatability of the dynamic Scheimpflug analyzer. Jpn J Ophthalmol.

[83] Chen K-J, Joda A, Vinciguerra R (2018). Clinical evaluation of a new correction algorithm for dynamic Scheimpflug analyzer tonometry before and after laser in situ keratomileusis and small-incision lenticule extraction. J Cataract Refract Surg.

[84] Lee H, Roberts CJ, Ambrósio R (2017). Effect of accelerated corneal crosslinking combined with transepithelial photorefractive keratectomy on dynamic corneal response parameters and biomechanically corrected intraocular pressure measured with a dynamic Scheimpflug analyzer in healthy myopic p. J Cataract Refract Surg.

[85] Fernández J, Rodriguez-Vallejo M, Martinez J (2017). New parameters for evaluating corneal biomechanics and intraocular pressure after small-incision lenticule extraction by Scheimpflug-based dynamic tonometry. J Cataract Refract Surg.

[86] Vinciguerra R, Romano V, Arbabi EM (2017). In vivo early corneal biomechanical changes after corneal cross-linking in patients with progressive KERATOCONUS. J Refract Surg.

[87] Śródka W (2009). Biomechanical model of human eyeball and its applications. Opt Appl.

[88] Kling S, Akca IB, Chang EW (2014). Numerical model of optical coherence tomographic vibrography imaging to estimate corneal biomechanical properties. J R Soc Interface.

[89] Shih P-J, Cao H-J, Huang C-J (2015). A corneal elastic dynamic model derived from Scheimpflug imaging technology. Ophthalmic Physiol Opt.

[90] Kadkhodaei M, Kasprzak H, Behrouz MJ (2018). Numerical and clinical investigation on the material model of the cornea in Corvis tonometry tests: differentiation between hyperelasticity and viscoelasticity. Mech Time-Depend Mater.

[91] Simonini I, Pandolfi A (2015). Customized finite element modelling of the human cornea. PLoS ONE.

[92] Simonini I, Angelillo M, Pandolfi A (2016). Theoretical and numerical analysis of the corneal air puff test. J Mech Phys Solids.

[93] Khan MA (2013). Numerical study on human cornea and modified multiparametric correction equation for Goldmann applanation tonometer. J Mech Behav Biomed Mater.

[94] Kling S, Bekesi N, Dorronsoro C (2014). Corneal viscoelastic properties from finite-element analysis of in vivo air-puff deformation. PLoS ONE.

[95] Sinha Roy A, Kurian M, Matalia H (2015). Air-puff associated quantification of non-linear biomechanical properties of the human cornea in vivo. J Mech Behav Biomed Mater.

[96] Ueki R, Maeda N, Fuchihata M (2018). Evaluation of corneal biomechanics in patients with keratectasia following LASIK using dynamic Scheimpflug analyzer. Jpn J Ophthalmol.

[97] Koprowski R, Tian L (2017). Quantitative assessment of the impact of blood pulsation on intraocular pressure measurement results in healthy subjects. J Ophthalmol.

[98] Danielewska ME, Iskander DR, Kowalska M (2012). Phase dependencies between longitudinal corneal apex displacement and cardiovascular signals: is the ocular pulse influenced by the electrical activity of the heart?. Clin Exp Optom.

[99] Goebels S, Eppig T, Wagenpfeil S (2017). Complementary keratoconus indices based on topographical interpretation of biomechanical waveform parameters : a supplement to established keratoconus indices. Comput Math Methods Med.

[100] McCafferty S, Lim G, Duncan W (2017). Goldmann tonometer error correcting prism: clinical evaluation. Clin Opthalmol.

[101] Wisse RPL, Peeters N, Imhof SM (2016). Comparison of Diaton transpalpebral tonometer with applanation tonometry in keratoconus. Int J Ophthalmol.

[102] Nakakura S (2018). Icare rebound tonometers: review of their characteristics and ease of use. Clin Ophthalmol.

[103] Fernandes P, Diaz-Rey JA, Queiros A (2005). Comparison of the ICare rebound tonometer with the Goldmann tonometer in a normal population. Ophthal Physiol Opt.

[104] Ittoop SM, Soohoo JR, Seibold LK (2016). Systematic review of current devices for 24-h intraocular pressure monitoring. Adv Ther.

[105] Turner MJ, Graham SL, Avolio AP (2013). Potential effects of systematic errors in intraocular pressure measurements on screening for ocular hypertension. Eye.

